# Current mutatome of SARS-CoV-2 in Turkey reveals mutations of interest

**DOI:** 10.3906/biy-2008-56

**Published:** 2021-02-09

**Authors:** Doğa ESKİER, Evren AKALP, Özlem DALAN, Gökhan KARAKÜLAH, Yavuz OKTAY

**Affiliations:** 1 İzmir Biomedicine and Genome Center (IBG), İzmir Turkey; 2 İzmir International Biomedicine and Genome Institute (iBG-İzmir), Dokuz Eylül University, İzmir Turkey; 3 Department of Medical Biology, Faculty of Medicine, Dokuz Eylül University, İzmir Turkey

**Keywords:** COVID-19, SARS-CoV-2, coronavirus, genome analysis, mutation profiling, Turkey, database

## Abstract

As the underlying pathogen for the COVID-19 pandemic that has affected tens of millions of lives worldwide, SARS-CoV-2 and its mutations are among the most urgent research topics worldwide. Mutations in the virus genome can complicate attempts at accurate testing or developing a working treatment for the disease. Furthermore, because the virus uses its own proteins to replicate its genome, rather than host proteins, mutations in the replication proteins can have cascading effects on the mutation load of the virus genome. Due to the global, rapidly developing nature of the COVID-19 pandemic, local demographics of the virus can be difficult to accurately analyze and track, disproportionate to the importance of such information. Here, we analyzed available, high-quality genome data of SARS-CoV-2 isolates from Turkey and identified their mutations, in comparison to the reference genome, to understand how the local mutatome compares to the global genomes. Our results indicate that viral genomes in Turkey has one of the highest mutation loads and certain mutations are remarkably frequent compared to global genomes. We also made the data on Turkey isolates available on an online database to facilitate further research on SARS-CoV-2 mutations in Turkey.

## 1. Introduction

Coronavirus disease 2019 (COVID-19) is an ongoing pandemic, characterized by long-term respiratory damage and slow onset fever, and caused by the SARS-CoV-2 betacoronavirus. The virus was first observed in human patients in late 2019, in the Wuhan province of China, and soon after showed the capacity for human-to-human transmission (Chan et al., 2020; Riou and Althaus, 2020). As of 17 August 2020 there are over 21 million confirmed cases and 767,158 recorded deathsWorld Health Organization (1948). WHO timeline – COVID-19 [online]. Website u2087 [accessed 17 August 2020].. In addition to the immediately apparent symptoms, its long term effect on humans are still topics of research (Kochi et al., 2020; Lee et al., 2020; Li et al., 2020; Zhu et al., 2020). Because COVID-19 is a highly transmissible disease with a capacity for asymptomatic transmission (Wong et al., 2020); understanding the disease, as well as its underlying pathogen and its routes of transmissions is a high priority topic. As a result, extant databases on viral pathogens, such as GISAIDThe GISAID Initiative (2008). GISAID [online]. Website u208b [accessed 27 August 2020]. (Elbe and Buckland‐Merrett, 2017) and NextstrainNextstrain (2015). Nextstrain [online]. Website u208d [accessed 27 August 2020]. (Hadfield et al., 2018) have become vital resources for researchers who seek to track the evolution of the virus during its transmissions.

SARS-CoV-2 has a single-stranded RNA genome that codes for the proteins responsible for its own replication, many of which are produced via cleavage of the Orf1ab polyprotein, the largest gene on the genome. Therefore, mutations in the SARS-CoV-2 genome can lead to cascading effects by reducing the fidelity of subsequent replication cycles. Key proteins in the RNA replication complex include nsps 7, 8, and 12 (also known as RNA dependent RNA polymerase or RdRp), which together form the core polymerase complex (Kirchdoerfer and Ward 2019; Peng et al., 2020), as well as nsp14, a dual function protein which joins the larger replication complex as a 3’-5’ error-correcting exonuclease (Subissi et al., 2014; Romano et al., 2020). Our previous findings show that frequently observed mutations in both nsp12 and nsp14 are associated with an increase in mutation density in the SARS-CoV-2 genome (Eskier et al., 2020a, 2020b, 2020c).

In this study, we aimed to analyze the current mutatome of SARS-CoV-2 in Turkey, with three main questions in mind: (i) are there any key reoccurring mutations observed in a large number of isolates? (ii) how does the distribution of mutations among isolates compare to other regions in the world? and finally, (iii) are there any mutations observed in Turkey but not the rest of the world? We focused on the latter two questions in particular, with an emphasis on mutations of interest previously described in the literature. Our findings reveal the presence of three main clades of SARS-CoV-2 in Turkey, roughly analogous to 19A, 20A, and 20B as described in NextStrain, with a preponderance of high mutability variants (Eskier et al., 2020a, 2020b, 2020c) compared to international isolates. Furthermore, we identified several frequently recurrent, previously uncharacterized variants in Turkey isolates not observed in isolates from other countries, which can serve as potential candidates for validation and study. Furthermore, we collected our analysis of Turkey isolates in a regularly maintained and updated database, which we hope will serve as a potential resource for future research on the local mutatome of SARS-CoV-2. 

## 2. Materials and methods

### 2.1. Genome sequence filtering, retrieval, and preprocessing

SARS-CoV-2 isolate genome sequences and the corresponding metadata were obtained from the GISAID EpiCoV database on 28 July 2020The GISAID Initiative (2008). EpiCoV database [online]. Website u208f [accessed 28 July 2020].. These sequences were filtered for location to limit our database to isolates with the location “Europe/Turkey”, which resulted in 180 isolate sequences. We applied further quality filters, including selecting only isolates obtained from human hosts (excluding environmental samples and animal hosts), those sequenced for the full length of the genome (sequence size of 29 kb or greater), and those with high coverage for the reference genome (<1% N content, < 0.05% unique mutations, no unverified indel mutations), which further narrowed down the list to 166 isolates. To ensure alignment accuracy, as characters that are not one of A, C, G, T, or N would not be aligned according to potential biological meanings of the alternative characters, all nonstandard unverified nucleotide masking was changed to N, using the Linux sed command, and the isolates were aligned against the SARS-CoV-2 reference genome using the MAFFT (v7.450) alignment software (Katoh et al., 2002). Variant sites in the isolates were annotated using snp-sites (2.5.1), bcftools (1.10.2)GitHub (2007). Bcftools repository [online]. Website u2093 [accessed 20 August 2020]., and ANNOVAR (release date 24 October 2019) software (Wang et al., 2010; Page et al., 2016), to identify whether a given mutation was synonymous or nonsynonymous. In addition, the 5’ untranslated region of the genome (bases 1–265) and the 100 nucleotides at the 3’ end were removed from the alignment and annotation files due to a high number of gaps and unidentified nucleotides.

### 2.2. Development of the database and user interfaces

The genome data is stored using the MariaDB 10.3.22 database installed on Debian Linux 10 operating system. For web application, the genome data is visualized on the map using jVectorMap with HTML 5 and Ajax web development techniques, using the Django 3.0.5. framework and Python 3.7.3 programming language. A modified version of TreeTime, an open-source phylogenetic analysis software, is used to create the phylogenetic tree (Sagulenko et al., 2018).

## 3. Results

### 3.1. The distributions of mutations across isolates in Turkey

Our analysis of the genome sequences of 166 isolates from Turkey revealed 258 distinct mutations across the isolates, 87 of which are observed in multiple isolates, and 43 of them are found in at least five isolates (hereafter referred to as recurring mutations). 19 of the 43 recurring mutations are nonsynonymous, 21 are synonymous, and 3 are found outside of coding regions. C>T transitions are the most common, comprising over half of the mutations, consistent with previous international findings on C>U hypermutations in SARS-CoV-2 (Simmonds, 2020). The most commonly seen mutations are 3037 C>T, 14408 C>T, and 23403 A>G, observed together in 139 of the isolates, with one singleton instance of 23403 A>G, also consistent with previous findings (Pachetti et al., 2020; Yin, 2020). Orf1ab mutations are the most common, comprising 23 of the recurring mutations, consistent with the size of the gene, as Orf1ab makes up two thirds of the SARS-CoV-2 genome. Orf9 (nucleocapsid; N) gene has the second highest number of recurring mutations (n = 7, however, 3 of them are block mutations of 28881–28883 trinucleotide), followed by Orf5 (membrane; M) and S genes (n = 5) (Table 1).

**Table 1:** Recurring mutations in Turkey.

Position	Reference	Variant	Frequency	Cities	Exonic function	Gene	Aminoacid change
23403	A	G	140	İstanbul (65), Karaman (1), Kastamonu (1), Nevşehir (2), Ankara (13), Kocaeli (5), Siirt (1), Aksaray (1), Sakarya (3), Afyon (1), Balıkesir (1), Konya (1), Denizli (2), Tekirdağ (1), Tokat (1), Kars (41)	Nonsynonymous SNV	S	p.D614G
3037	C	T	139	İstanbul (65), Karaman (1), Kastamonu (1), Nevşehir (2), Ankara (13), Kocaeli (4), Siirt (1), Aksaray (1), Sakarya (3), Afyon (1), Balıkesir (1), Konya (1), Denizli (2), Tekirdağ (1), Tokat (1), Kars (41)	Synonymous SNV	ORF1a; ORF1ab	p.F924F
14408	C	T	139	İstanbul (65), Karaman (1), Kastamonu (1), Nevşehir (2), Ankara (13), Kocaeli (4), Siirt (1), Aksaray (1), Sakarya (3), Afyon (1), Balıkesir (1), Konya (1), Denizli (2), Tekirdağ (1), Tokat (1), Kars (41)	Nonsynonymous SNV	ORF1ab	p.P4715L
241	C	T	121	İstanbul (66), Nevşehir (1), Kocaeli (3), Ankara (6), Denizli (1), Ağrı (1), Tekirdağ (1), Tokat (1), Kars (41)		ORF1a; ORF1ab	
28881	G	A	73	İstanbul (40), Sakarya (2), Kocaeli (3), Ankara (3), Kars (25)	Nonsynonymous SNV	ORF9	p.R203K
28882	G	A	73	İstanbul (40), Sakarya (2), Kocaeli (3), Ankara (3), Kars (25)	Synonymous SNV	ORF9	p.R203R
28883	G	C	73	İstanbul (40), Sakarya (2), Kocaeli (3), Ankara (3), Kars (25)	Nonsynonymous SNV	ORF9	p.G204R
25563	G	T	61	İstanbul (24), Karaman (1), Kastamonu (1), Nevşehir (2), Ankara (8), Kocaeli (1), Siirt (1), Aksaray (1), Sakarya (1), Afyon (1), Balıkesir (1), Konya (1), Tekirdağ (1), Tokat (1), Kars (16)	Nonsynonymous SNV	ORF3a	p.Q57H
18877	C	T	58	İstanbul (24), Kastamonu (1), Nevşehir (2), Ankara (7), Kocaeli (1), Siirt (1), Aksaray (1), Sakarya (1), Afyon (1), Balıkesir (1), Konya (1), Tekirdağ (1), Tokat (1), Kars (15)	Synonymous SNV	ORF1ab	p.L6205L
7765	C	T	35	İstanbul (18), Siirt (1), Nevşehir (1), Tekirdağ (1), Kars (14)	Synonymous SNV	ORF1a; ORF1ab	p.S2500S
17690	C	T	35	İstanbul (18), Siirt (1), Nevşehir (1), Tekirdağ (1), Kars (14)	Nonsynonymous SNV	ORF1ab	p.S5809L
11083	G	T	31	Kayseri (1), İstanbul (11), Karaman (1), Ankara (6), Balıkesir (1), Çanakkale (1), Eskişehir (1), Kocaeli (4), Mardin (1), Ağrı (1), Kars (3)	Nonsynonymous SNV	ORF1a; ORF1ab	p.L3606F
29742	G	T	24	Kayseri (1), Ankara (5), Kocaeli (5), Balıkesir (1), Çanakkale (1), Eskişehir (1), Mardin (1), İstanbul (8), Ağrı (1)		ORF10;ORF9	
1397	G	A	23	Kayseri (1), Ankara (5), Balıkesir (1), Çanakkale (1), Eskişehir (1), Kocaeli (4), Mardin (1), İstanbul (8), Ağrı (1)	Nonsynonymous SNV	ORF1a; ORF1ab	p.V378I
12809	C	T	23	İstanbul (20), Kars (3)	Nonsynonymous SNV	ORF1a; ORF1ab	p.L4182F
28688	T	C	23	Kayseri (1), Ankara (5), Balıkesir (1), Çanakkale (1), Eskişehir (1), Kocaeli (4), Mardin (1), İstanbul (8), Ağrı (1)	Synonymous SNV	ORF9	p.L139L
27703	G	T	20	Kars (20)	Nonsynonymous SNV	ORF7a	p.V104F
24262	G	T	20	Kars (20)	Nonsynonymous SNV	S	p.M900I
313	C	T	19	Kars (19)	Synonymous SNV	ORF1a; ORF1ab	p.L16L
2509	C	T	19	Kars (19)	Synonymous SNV	ORF1a; ORF1ab	p.P748P
13620	C	T	19	Kars (19)	Synonymous SNV	ORF1ab	p.D4452D
14724	C	T	19	Kars (19)	Synonymous SNV	ORF1ab	p.F4820F
19839	T	C	18	Ankara (2), İstanbul (15), Kars (1)	Synonymous SNV	ORF1ab	p.N6525N
26735	C	T	15	Kastamonu (1), Nevşehir (1), Ankara (5), Kocaeli (1), Aksaray (1), Sakarya (1), Afyon (1), Balıkesir (1), Konya (1), İstanbul (2)	Synonymous SNV	ORF5	p.Y71Y
8326	C	T	14	Kars (14)	Synonymous SNV	ORF1a; ORF1ab	p.D2687D
2113	C	T	13	İstanbul (11), Nevşehir (1), Tekirdağ (1)	Synonymous SNV	ORF1a; ORF1ab	p.I616I
884	C	T	12	Balıkesir (1), Çanakkale (1), Eskişehir (1), Kocaeli (3), Ankara (3), İstanbul (3)	Nonsynonymous SNV	ORF1a; ORF1ab	p.R207C
8653	G	T	12	Balıkesir (1), Çanakkale (1), Eskişehir (1), Kocaeli (3), Ankara (3), İstanbul (3)	Nonsynonymous SNV	ORF1a; ORF1ab	p.M2796I
26549	C	T	12	Kocaeli (2), İstanbul (5), Ankara (3), Tokat ( 1), Denizli (1)	Synonymous SNV	ORF5	p.T9T
5015	G	A	11	Kars (11)	Nonsynonymous SNV	ORF1a; ORF1ab	p.V1584M
28854	C	T	10	Ankara (3), Aksaray (1), Sakarya (1), Konya (1), İstanbul (3), Kars (1)	Nonsynonymous SNV	ORF9	p.S194L
228	C	T	9	Kocaeli (1), İstanbul (4), Ankara (3), Denizli (1)		ORF1a; ORF1ab	
22444	C	T	9	Ankara (3), Aksaray (1), Sakarya (1), Konya (1), İstanbul (3)	Synonymous SNV	S	p.D294D
9514	A	G	8	Kocaeli (1), İstanbul (4), Ağrı (1), Ankara (2)	Synonymous SNV	ORF1a; ORF1ab	p.L3083L
26720	G	C	8	Kocaeli (1), İstanbul (4), Ağrı (1), Ankara (2)	Synonymous SNV	ORF5	p.V66V
9479	G	T	7	Kocaeli (1), İstanbul (4), Ağrı (1), Ankara (1)	Nonsynonymous SNV	ORF1a;ORF1ab	p.G3072C
28835	T	C	7	Kocaeli (1), İstanbul (4), Ağrı (1), Ankara (1)	Nonsynonymous SNV	ORF9	p.S188P
5736	C	T	6	Ankara (3), Denizli (1), İstanbul (2)	Nonsynonymous SNV	ORF1a; ORF1ab	p.A1824V
16428	C	T	6	Kars (6)	Synonymous SNV	ORF1ab	p.Y5388Y
25611	C	A	6	İstanbul (6)	Synonymous SNV	ORF3a	p.L73L
28857	G	T	6	Kars (6)	Nonsynonymous SNV	ORF9	p.R195I
10702	C	T	5	Eskişehir (1), Ankara (1), İstanbul (3)	Synonymous SNV	ORF1a; ORF1ab	p.D3479D
20268	A	G	5	Ankara (2), Denizli (1), Kocaeli (1), İstanbul (1)	Synonymous SNV	ORF1ab	p.L6668L

To identify which of the recurring mutations are stronger indicators of Turkey genotype, we compared their frequency in the isolate population from Turkey to frequencies in other geographical regions, using a metric of mutation instance per sequenced isolate. To eliminate the potential confounding effect of earlier isolates having a lower number of mutations on average, and different regions having started sequencing efforts in different timetables, we selected isolates sequenced after the day when each region of interest had at least ten isolates sequenced. As Turkey was the latest region to have the required number of isolates (19 March 2020), it was used as the filtering metric. Four of the recurring mutations were found only in Turkey isolates, and six more were not recurring mutations in other regions. Therefore, we focused the comparisons on the remaining 33 recurring mutations. We identified the percentages of these mutations in each region (Africa, Asia, Europe, North America, Oceania, South America), as well as worldwide totals, excluding isolates from Turkey, where applicable, and compared them to the corresponding percentages in Turkey (Table 2). With two exceptions (241 C>T and 20268 A>G), all of the mutations have higher percentages in Turkey compared to worldwide percentages. In addition, isolates from Turkey comprise over 50% of the isolates worldwide carrying six of the mutations (228 C>T, 8326 C>T, 12809 C>T, 13620 C>T, 14724 C>T, and 27703 C>T), and 20 of the mutations have higher percentages in Turkey than any other region. Among these six mutations, 228 C>T was first detected in Canada on March 11 and in Turkey (İstanbul) on March 18China National Center for Bioinformation (2009). 2019 Novel Coronavirus Resource (2019nCoVR) [online]. Website https://bigd.big.ac.cn/ncov/variation/annotation/ [accessed 28 August 2020].. The 8326 C > T mutation was first isolated from a patient in Taipei on March 19 and only two other cases was reported (UK and Denmark) before the first case in Turkey (Kars) on April 29. Thirteen more cases with 8326 C>T in the same city implicates local transmission, and with 8 cases reported on the most recent update (July 15), this particular mutation is a candidate for even further spread, assuming that the isolates sequenced were randomly selected across the infected population, instead of all being selected from a known cluster of patients. The 12809 C>T mutation was first reported in an isolate from Washington/USA collected on March 14, which spread to five more states by the end of the month; however, only 4 more cases were reported afterwards, the last being one on April 27. The first isolate from Turkey with 12809 C>T mutation was collected on April 13 in İstanbul (EPI_ISL_480230). Being present in only three other countries with one isolate each (UK, India, Australia), in addition to the USA, this mutation was very likely an introduction from the USA. Since then, 20 more cases in İstanbul and 3 more cases in Kars were reported, all in April and May. 13620 C>T mutation was first reported in an isolate in Italy on March 5 and later in South Africa, USA, Denmark, Belgium, Luxembourg and Singapore by April 6. However, only three more cases were reported for the rest of April: two in Italy on April 12, and one in USA on April 28. The first case with the same mutation in Turkey was reported in Kars on May 17 and has been reported in 18 more cases, all in the same city. Initial phylogenetic analysis does not support introduction of this mutation from abroad, however, limited sampling makes it difficult to reach a definitive conclusion. 13620 C>T, 14724 C>T, and 27703 G>T mutations are linked in SARS-CoV-2 genomes from Turkey, all from a single city (Kars), suggesting a founder effect and local transmission. It bears noting that each of the four mutations exclusively found as recurring in Turkey are limited to a single batch of isolates obtained by a single center, therefore pending verification.

**Table 2:** Frequencies of SARS-CoV-2 mutations in different geographical regions.

Mutation	Africa	Asia	Europe	North America	Oceania	South America	Turkey	Worldwide
23403A>G	88.73% (252)	58.15% (835)	82.34% (10484)	78.15% (4470)	67.07% (894)	93.48% (258)	84.34% (140)	78.94% (17193)
3037C>T	79.58% (226)	58.84% (845)	82.03% (10444)	78.25% (4476)	66.99% (893)	93.48% (258)	83.73% (139)	78.7% (17142)
14408C>T	87.68% (249)	58.57% (841)	82.32% (10481)	78.36% (4482)	67.14% (895)	93.48% (258)	83.73% (139)	79% (17206)
241C>T	88.73% (252)	58.29% (837)	82.24% (10471)	77.36% (4425)	51.24% (683)	93.48% (258)	72.89% (121)	77.71% (16926)
28881G>A	22.18% (63)	16.92% (243)	38.73% (4931)	5.03% (288)	13.5% (180)	48.55% (134)	43.98% (73)	26.81% (5839)
28882G>A	22.18% (63)	16.85% (242)	38.68% (4925)	5.02% (287)	13.28% (177)	48.55% (134)	43.98% (73)	26.76% (5828)
28883G>C	22.18% (63)	16.92% (243)	38.67% (4924)	5.02% (287)	13.35% (178)	48.55% (134)	43.98% (73)	26.76% (5829)
25563G>T	10.21% (29)	26.46% (380)	12.21% (1554)	65.93% (3771)	28.21% (376)	30.8% (85)	36.75% (61)	28.44% (6195)
18877C>T	2.46% (7)	15.67% (225)	1.2% (153)	5.38% (308)	1.73% (23)	9.78% (27)	34.94% (58)	3.41% (743)
7765C>T	1.06% (3)	0.35% (5)	0.57% (73)	0.07% (4)	0.15% (2)	0% (0)	21.08% (35)	0.4% (87)
17690C>T	1.06% (3)	0.14% (2)	0.47% (60)	0.07% (4)	0.08% (1)	0% (0)	21.08% (35)	0.32% (70)
11083G>T	8.8% (25)	28.41% (408)	10.91% (1389)	3.22% (184)	15.68% (209)	5.07% (14)	18.67% (31)	10.23% (2229)
29742G>T	0.7% (2)	2.44% (35)	0.13% (17)	0.24% (14)	3.45% (46)	0% (0)	14.46% (24)	0.52% (114)
1397G>A	1.06% (3)	2.44% (35)	0.03% (4)	0.21% (12)	4.43% (59)	0% (0)	13.86% (23)	0.52% (113)
12809C>T	0% (0)	0% (0)	0.02% (3)	0.12% (7)	0% (0)	0% (0)	13.86% (23)	0.05% (10)
28688T>C	1.06% (3)	2.51% (36)	0.02% (3)	0.14% (8)	4.35% (58)	0% (0)	13.86% (23)	0.5% (108)
27703G>T	0% (0)	0% (0)	0.06% (8)	0% (0)	0.08% (1)	0% (0)	12.05% (20)	0.04% (9)
313C>T	0.7% (2)	4.81% (69)	1.56% (199)	0.72% (41)	0.75% (10)	0.36% (1)	11.45% (19)	1.48% (322)
13620C>T	0% (0)	0% (0)	0.04% (5)	0.03% (2)	0% (0)	0% (0)	11.45% (19)	0.03% (7)
14724C>T	0% (0)	0% (0)	0.07% (9)	0.12% (7)	0.08% (1)	0.36% (1)	11.45% (19)	0.08% (18)
19839T>C	0.7% (2)	0.63% (9)	2.37% (302)	0.59% (34)	0.38% (5)	0.36% (1)	10.84% (18)	1.62% (353)
26735C>T	0.35% (1)	14.35% (206)	0.57% (73)	0% (0)	0.6% (8)	0% (0)	9.04% (15)	1.32% (288)
8326C>T	0% (0)	0.07% (1)	0.03% (4)	0% (0)	0% (0)	0% (0)	8.43% (14)	0.02% (5)
2113C>T	0.7% (2)	0.14% (2)	0.49% (62)	0.03% (2)	0.08% (1)	0% (0)	7.83% (13)	0.32% (69)
884C>T	0.7% (2)	1.95% (28)	0.02% (3)	0.1% (6)	0.38% (5)	0% (0)	7.23% (12)	0.2% (44)
8653G>T	0.7% (2)	1.88% (27)	0.04% (5)	0.12% (7)	0.3% (4)	0% (0)	7.23% (12)	0.21% (45)
28854C>T	0.7% (2)	6.69% (96)	2.52% (321)	1.38% (79)	0.83% (11)	0.36% (1)	6.02% (10)	2.34% (510)
228C>T	0.35% (1)	0.07% (1)	0.02% (3)	0% (0)	0.08% (1)	0% (0)	5.42% (9)	0.03% (6)
22444C>T	0% (0)	6.48% (93)	0.02% (2)	0% (0)	0% (0)	0% (0)	5.42% (9)	0.44% (95)
9479G>T	0.35% (1)	0.14% (2)	0.07% (9)	0.05% (3)	0.15% (2)	0% (0)	4.22% (7)	0.08% (17)
16428C>T	0% (0)	0% (0)	0.01% (1)	0.12% (7)	0% (0)	0.36% (1)	3.61% (6)	0.04% (9)
28857G>T	0% (0)	0.07% (1)	0.09% (12)	0.02% (1)	0% (0)	0% (0)	3.61% (6)	0.06% (14)
20268A>G	3.17% (9)	1.04% (15)	8.18% (1042)	1.03% (59)	4.13% (55)	9.06% (25)	3.01% (5)	5.53% (1205)

Afterwards, we sought to understand how the mutation load of the isolates in Turkey compare to distributions in other regions. Using our previous date filter, we calculated the number of single nucleotide variants (SNVs) per isolate in each region (Table 3). Turkey had the highest number of SNVs per isolate, followed by South America. In comparison, Africa, another region which started sequencing efforts later than the other regions, had a mean SNV number lower than that of Asia, the region with the earliest sequences available, implying that the mutation numbers are strongly influenced by other factors in addition to the date of introduction of the virus to the region. We also compared the number of SNVs per isolate in each region per gene, normalized by kilobase of gene region (Table 4). Turkey had the most SNVs of any region in Orf1ab, M, and Orf7a genes, with Orf7a having more than three times as many SNVs as any other region.

**Table 3:** Mean number of variants per isolate in different geographical regions.

Region	Orf1ab	S	Orf3	E	M	Orf6	Orf7a	Orf7b	Orf8	N	Orf10
Turkey	0.25	0.32	0.49	0.06	0.38	0.03	0.44	0.05	0.11	1.45	0.06
Worldwide	0.19	0.29	0.62	0.08	0.13	0.11	0.11	0.10	0.38	0.84	0.10
Africa	0.21	0.40	0.25	0.06	0.15	0.00	0.06	0.00	0.23	0.75	0.12
Asia	0.20	0.31	0.48	0.11	0.31	0.10	0.13	0.12	0.36	0.83	0.10
Europe	0.18	0.29	0.48	0.09	0.13	0.09	0.12	0.09	0.15	1.10	0.11
North America	0.20	0.26	0.97	0.06	0.09	0.12	0.08	0.12	0.84	0.27	0.11
Oceania	0.19	0.26	0.66	0.05	0.13	0.08	0.07	0.05	0.62	0.75	0.06
South America	0.16	0.33	0.59	0.08	0.06	1.68	0.11	0.14	0.11	1.46	0.06

**Table 4:** SNV densities of SARS-CoV-2 genes in different geographical regions.

Region	Mean number ofvariants per isolate
Turkey	10.18
Worldwide	8.01
Africa	8.47
Asia	8.53
Europe	7.88
North America	8.20
Oceania	7.54
South America	9.32

### 3.2. Database implementation

Data regarding Turkey isolates are available as a database comprising an interactive phylogenetic tree of the isolates, a geographical heatmap of sequenced isolates, and tables for both the mutatome of individual isolates, and summaries of the mutations observed in the isolates (Figure). The phylogenetic tree can be viewed both in real time and divergence time, and colored according to nucleotide of interest, location, or sequencing date. The tables are generated using the sequencing metadata available from GISAID as well as ANNOVAR variant annotation tables. We aim to regularly validate and update the database as new sequences are made availableThe database is freely accessible at u2095.. Future plans include implementation of Nextstrain clade and branch information in the phylogenetic tree to aid the user in comparisons with international sequencing data.

**Figure 1:**
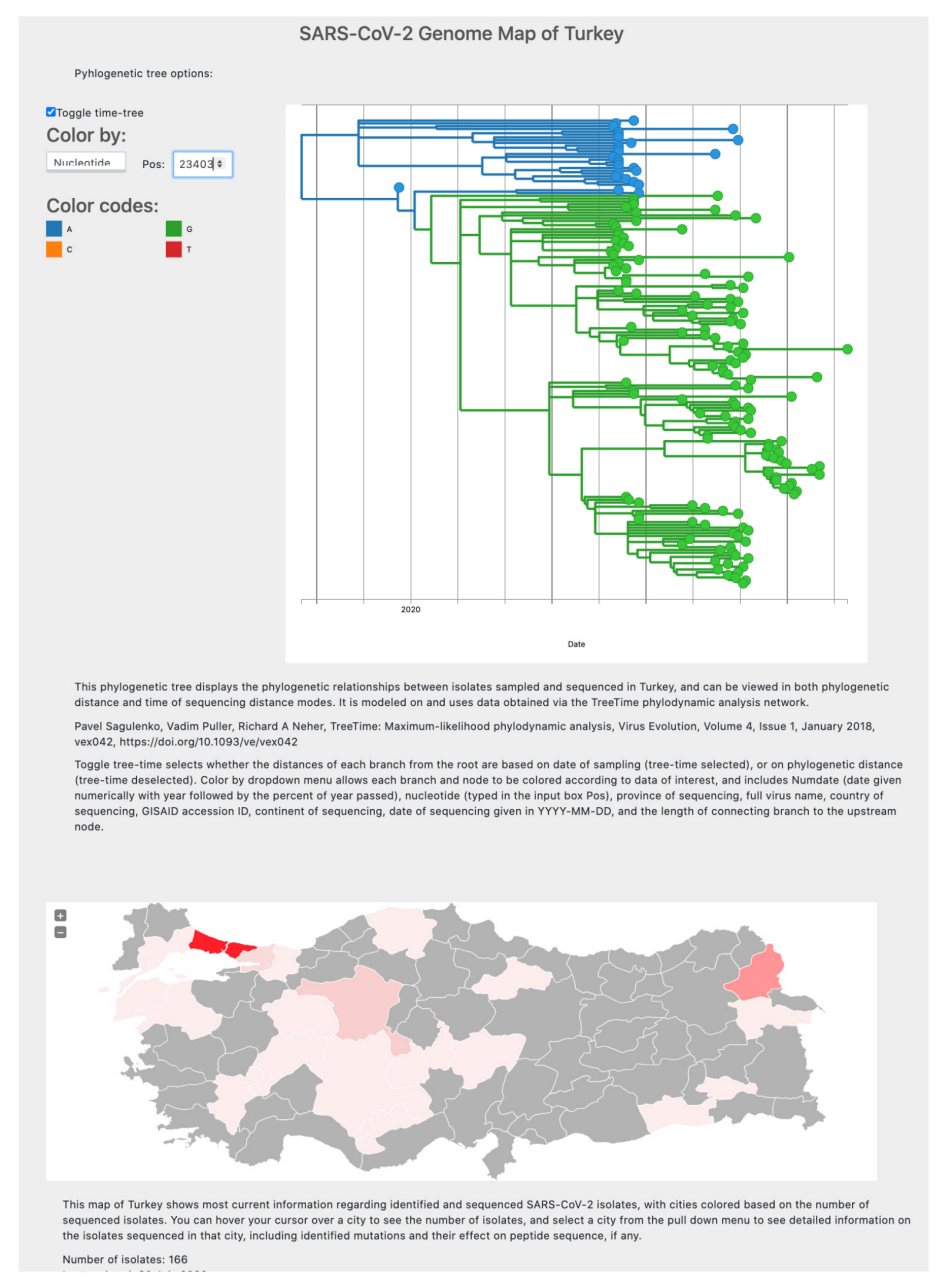
Snapshot of SARS-CoV-2 genome map of Turkey database. Due to size constraints, tables showing information on individual isolates, or summaries of individual variants, are not included.

## 4. Discussion

COVID-19 has been causing tremendous challenges for clinicians, healthcare systems, societies, and governments, and has required development of novel approaches to fight the pandemic. With an unpredictable future course for the ongoing pandemic, close monitoring and characterization of mutations has emerged as top priorities for better understanding of possible genotype-phenotype relations, and therefore better management of healthcare efforts. 

Mutations in any viral infection, especially those that have crossed interspecies barriers, have to be considered in the context of natural selection. As the evolution of a virus will likely affect its fitness in a new host, any attempts against such an infection have to consider the causal relationships between genomic variances and the spread of the virus. Previous studies suggest that the selective pressure on mutations in SARS-CoV-2 in human hosts are largely confined to modest positive selection, with very little purifying selection, due to the short span of the pandemic, and that most of the positive selection have occurred in previous hosts (MacLean et al., 2020). Therefore, any investigation of the mutations will need to consider most of the mutations have to be beneficial or neutral to create true strains of the virus. A comprehensive analysis by Jungreis et al. (2020) showed that SARS-Cov-2 mutations are excluded from the evolutionarily conserved amino acid residues and nucleotides, and the authors concluded both synonymous and nonsynonymous mutations are under purifying selection. Therefore, not only the nonsynonymous mutations, but also the synonymous ones should be considered as potentially functional.

Many studies already provided lines of evidence that supports a role for the S D614G mutation in increased infectivity and likely in transmissibility of SARS-CoV-2 (Daniloski et al., 2020; Korber et al., 2020). It is possible that new mutations that affect viral behavior may arise, and therefore emergence and spreading of such mutations should be monitored closely. However, with tens of millions affected worldwide, monitoring of every single mutation is a challenging task. We believe that our database will provide a valuable and practical resource for researchers in Turkey, as well as in other countries, to track the spread of SARS-CoV-2 mutations in Turkey.

Our findings show the viral isolates in Turkey have accumulated a higher number of mutations compared to other regions on average, even after normalizing for the isolates sequenced earlier during the pandemic having accumulated fewer mutations. Furthermore, it has more mutations in the Orf1ab gene, which produces the polyprotein that is cleaved into the mature peptides responsible for viral replication, than any other region. In addition, it has the third highest number of mutations in the S gene, which is responsible for the viral infection of the cells. As these two genes have the highest potential impact on the replication and transmission cycle of the virus, a higher mutation density in these genes can lead to an accelerated mutation rate. Of note, the 18877 C>T mutation in nsp14, the 3’-5’ exonuclease responsible for error correction during genomic replication, has the second highest frequency in Turkey of any country6. Our previous study (Eskier et al., 2020a) shows a strong correlation between increased mutation density and the 18877 C>T mutation, which might be a potential reason for Turkey’s increased SNV average per isolate.

Two groups of mutations we identified that is worth further attention are the 3037 C>T, 14408 C>T, 23403 A>G haplotype, and the 28881–28883 block mutation. Both of these groups of mutations are found almost exclusively together, both in Turkey, and worldwide. In both cases, Turkey has a higher incidence of mutations in these groups than worldwide averages, and four of the major regions (Asia, Europe, North America, Oceania). We previously found that the 14408 C>T and 23403 A>G mutations,when occurring together, are strongly associated with increased mutation density over time (Eskier et al., 2020a), and the prevalence of both these mutations and the 18877 C>T mutation in Turkey isolates may further contribute to a variant-rich mutation landscape (Eskier et al., 2020b). 28881–28883 GGG>AAC is found on the N gene, whose product is responsible for packaging the genome into newly produced virions in cells, and regulating host cell response (McBride et al., 2014). The mutation disrupts an SR-rich motif in the nucleocapsid protein, which was found to cause reduced transmissibility in SARS-CoV, a similar betacoronavirus with high homology to SARS-CoV-2 (Tylor et al., 2009; Ayub, 2020). It is not clear whether the mutation groups are selected together and show homoplasic recurrence across isolates, or if they are a result of strong founder effect. 

A major concern when analyzing the isolate sequences from Turkey is the limited nature of the data. The sequences are few in number, and their geographical and temporal distributions are highly skewed, leading to difficulty in understanding the transmission routes of the virus across the country. Furthermore, new sequences are often made available in large batches by the centers, which further introduces bias to the samples by potentially generating sequencing or assembly artifacts to the sequences. Unless verified by multiple centers, in multiple batches, or by other experimental methods, caution is required when studying these mutations. As more genomes are sequenced, a more clear picture of the SARS-CoV-2 mutatome in Turkey will emerge and we will likely be able to draw more solid conclusions. 

Finally, it should be noted that mutational profiles of viral genomes may determine whether infected patients will develop lasting immunity and remain protected from re-infection. Although exposure to SARS-CoV-2 protected rhesus macaques from re-infection with the same strain of virus (Deng et al., 2020), there are questions still remaining to be answered related to whether each recovered patient will have lasting immunity. Recent news within days reported that four patients from Hong Kong, Belgium, the Netherlands, and USA, who had earlier recovered from COVID-19 has been reinfected, with a different strain of SARS-CoV-2 than the original infectionEuronews (1993). Euronews [online]. Website u2098 [accessed 28 August 2020]. (Tillett et al., 2021). In support of this observation, an earlier study reported that convalescent plasma from some of the COVID-19 patients showed reduced neutralizing activity against pseudoviruses with D614G mutation in culture environment (Hue et al., 2020). We do not have a clear understanding of the viral determinants of lasting immunity to SARS-CoV-2, however, it seems that certain viral proteins may be more critical than others, based on analyses of patient plasma samples. Grifoni et al. (2020) suggested that M, Spike and N proteins are the major determinants of CD4+ response, with additional responses to nsp3, nsp4, ORF3a and ORF8. Hachim et al. (2020) showed that ORF8, ORF3b and N proteins of SARS-CoV-2 elicited the strongest specific antibody responses in infected patients. It is plausible that certain mutations within these proteins affect the immune response, however, it remains to be explored whether any of the mutations common or more frequently seen in Turkish isolates have any effect on the immune response. 
